# 4571 Examination of the relationship between age, program duration and risk profiles among sex-trafficked youth in a specialty court

**DOI:** 10.1017/cts.2020.267

**Published:** 2020-07-29

**Authors:** Mekeila Cook

**Affiliations:** 1 Vanderbilt University Medical Center

## Abstract

OBJECTIVES/GOALS: Youth experience worse health and behavioral outcomes the longer they are in the juvenile justice system. This study examines whether age at entry and length of time in a specialty juvenile court program predicts citations, bench warrants, and running away among sex-trafficked girls. METHODS/STUDY POPULATION: Domestic minor sex trafficking (DMST) is exploitation and abuse of children for commercial sexual purposes in exchange for money or other goods/services. Historically, the response to DMST has been punitive, resulting in youth being cited and detained for offenses like prostitution. The specialty court offers enhanced physical/mental health services to trafficked youth. Data come from case files in the specialty court for program participants from 2012-2014 (N = 184). Descriptive, bivariate, and Poisson regression analyses were performed to examine risk profile measures: bench warrants, citations, running away, and foster placements. RESULTS/ANTICIPATED RESULTS: All were (cis)female, 74% were African-American, 96%, US citizens, with average age of 16. Participants lived in approximately 4.5 group homes or foster placements prior to program entry; 56% of youth had run away. Youth also averaged nearly two bench warrants before specialty court participation. Bivariate analysis indicates older age at entry into juvenile court was associated with fewer episodes of running away (*p*<.02) and new citations (*p*<.001). Poisson regression estimated older age at entry into the juvenile justice system was associated with fewer bench warrants, citations, foster placements, but not running away while in the program. Additionally, longer duration between time at first citation and entry into the program was associated with fewer bench warrants, running away, and citations. DISCUSSION/SIGNIFICANCE OF IMPACT: Younger girls may be particularly vulnerable to trafficking and recidivism without early and persistent intervention. Youth experiencing sex trafficking need to be diverted away from juvenile justice to comprehensive trauma informed services.

